# Inflammatory responses to dietary and surgical weight loss in male and female mice

**DOI:** 10.1186/s13293-019-0229-7

**Published:** 2019-04-03

**Authors:** Cameron Griffin, Chelsea R. Hutch, Simin Abrishami, Daria Stelmak, Leila Eter, Ziru Li, Eric Chang, Devyani Agarwal, Brian Zamarron, Mita Varghese, Perla Subbaiah, Ormond A. MacDougald, Darleen A. Sandoval, Kanakadurga Singer

**Affiliations:** 1 Department of Pediatrics, University of Michigan Medical School, Ann Arbor, MI USA; 2 Department of Surgery, University of Michigan Medical School, Ann Arbor, MI USA; 3 Department Molecular & Integrative Physiology, University of Michigan Medical School, Ann Arbor, MI USA; 4 Department of Mathematics and Statistics, Oakland University, Rochester, MI 48309 USA; 5 Department of Pediatrics, Division of Pediatric Endocrinology, D1205 MPB, 1500 E Medical Center Dr., Ann Arbor, MI 48109 USA

**Keywords:** Obesity, Macrophage, Myelopoiesis, Metabolism, Sex-differences, Bariatric surgery

## Abstract

**Background:**

Weight loss by surgery or lifestyle changes is strongly recommended for obese individuals to improve metabolic health, but the underlying impairments that persist from a history of obesity remain unclear. Recent investigations demonstrate a persistent inflammatory state with weight loss and bariatric surgery, but the mechanism and impact are not fully understood. Additionally, these studies have not been performed in females although women are the majority of individuals undergoing weight loss interventions.

**Methods:**

The goal of this study was to determine the sex differences in metabolically induced inflammation after dietary weight loss (WL) or bariatric surgery. Following a 60% high-fat diet (HFD) for 12 weeks, C57Bl/6j mice underwent either a dietary switch to normal chow for WL or vertical sleeve gastrectomy (VSG) and were evaluated 8 weeks after intervention. WL effects on myelopoiesis were further evaluated with bone marrow chimeras.

**Results:**

Both sexes had a decrease in adiposity and total weight following WL or VSG intervention. With HFD, females had very little inflammation and no further increase with WL, but males had persistent inflammation even after WL despite metabolic improvement. Interestingly, after VSG, myeloid inflammation was increased in the livers of males and to a lesser extent in females.

**Conclusions:**

These studies demonstrate that regardless of sex, it is critical to assess an individuals’ history of obesity rather than just rely on current weight status in medical decision-making. There are long-lasting effects on tissue inflammation in both sexes especially with surgical weight loss. Dietary change is overall most effective to improve meta-inflammation in obese males on its own or in combination with surgical weight loss.

## Introduction

Obesity is a significant public health crisis due to the rise in predominantly metabolic sequelae [1–3]. Many weight loss interventions have been created to attenuate the medical sequelae of obesity. While there have been pharmacologic treatments [4], the most popular sought-after interventions include dietary/lifestyle changes and more recently bariatric surgery [5]. Although a 10–20% reduction in weight can have significant metabolic improvements with improved glucose and insulin levels [6], effects on the chronic inflammatory state of obesity have been less thoroughly examined.

Following high-fat caloric intake, adipose tissue depots expand leading to obesity. Concurrent with this expansion is an increase of both resident and recruited tissue macrophages in response to adipocyte hypertrophy [7]. An increase in chemokine signals within gonadal white adipose tissue (GWAT), the main visceral fat depot in animal models, leads to expansion of recruited macrophages. Specifically, monocyte chemoattractant protein-1/C-C chemokine receptor type 2 (MCP-1/CCR2) signals are known to recruit Ly6c^hi^ monocytes, which differentiate into CD64^+^/CD11c^+^ adipose tissue macrophages (CD11c^+^ ATMs) [7] and accumulate in crown-like structures (CLS), around damaged and dying adipocytes [8]. CD11c^+^ ATMs are linked to obesity-related sequelae and are functionally distinct from resident, CD64^+^CD11c^−^ macrophages (CD11c^−^ ATMs) [9]. While these CD11c^+^ macrophages typically help with maintaining tissue homeostasis [10], the expansion and detrimental cytokine production leads to a pathologic state of tissue dysfunction.

Weight loss has become a primary recommendation for resolution of obesity and obesity-related diseases. However, recent investigations into the impact of weight loss in animal models have determined that even with weight loss, there is an underlying myeloid inflammation that remains for at least 8 weeks [11]. Similar findings have been observed in studies using vertical sleeve gastrectomy, demonstrating a persistent inflammatory state post-weight loss [12, 13]. While these are clinically relevant pre-clinical findings, there is a critical gap in the inclusion of female animal models in these studies. This is an important gap to fill given that over 80% of bariatric surgeries [14, 15] and a majority of dietary and exercise weight loss programs are predominantly filled with women [16]. Also, there is some controversy in the results since some studies have demonstrated improvement in systemic inflammation with dietary weight loss [17, 18] and some report no changes at all within adipose tissue [19, 20]. These contradictory results may be due to the age, timing of weight loss, and sex differences.

In order to understand if the persistent tissue inflammation and metabolic dysfunction is a critical issue in men and women after weight loss, we have pursued investigations in both male and female animal models. We hypothesized that given the priming of males to meta-inflammation, persistent inflammation would be most prominent in male mice than females with obesity and weight loss regardless of method. Our studies show that male animals had improved but some persistent adipose tissue and liver inflammation even after weight loss while female adipose tissue remained in a low-inflammatory state. The programming of this priming for tissue inflammation persisted after bone marrow transplantation (BMT) in male animals. Surprisingly, with vertical sleeve gastrectomy, adipose tissue inflammation due to resident CD11c^−^ macrophages was sustained in both post-surgical males and females.

## Methods

### Animals and animal care

Male and female C57Bl/6J mice were purchased from Jackson Laboratory (Bar Harbor, Maine). Six-week-old male and female mice were fed either a normal diet (LabDiet 13.5% fat 5L0D, St. Louis, MO) or high-fat diet (HFD, Research Diets D12492 60% fat, New Brunswick, NJ). A subgroup of HFD-induced obese mice was switched back to ND after 12 weeks of HFD to create the weight loss (WL) animal model. About 4–8 mice were used for dietary experiments. Glucose tolerance tests (GTT) were performed after 6 h of fasting. Mice were injected with intraperitoneal insulin (0.7 g/kg) and D-glucose (Gibco, Indianapolis, IN) measured by Free-Style Lite glucose meter. All mouse procedures were approved by the University of Michigan Committee on Use and Care of Animals and were conducted in compliance with the Institute of Laboratory Animal Research Guide for the Care and Use of Laboratory Animals.

### Vertical sleeve gastrectomy surgery

Animals were placed on 12 weeks of HFD starting at 6 weeks of age. Two weeks prior to surgery (after 10 weeks of HFD), mice were singly housed. Mice were then separated into two surgery groups, sham surgery or vertical sleeve gastrectomy (VSG). After surgery, animals from the sham group were either continued on 60% high-fat diet for 8 weeks or switched to control chow. All surgeries were performed under isoflurane anesthesia as previously described [12, 21]. For VSG, the lateral stomach was resected along the major curvature to form a remnant tube. A *N* of 4–8 again was used in sham groups but a larger *N* was used in the VSG groups for experiments (8–12) given complications that could occur in this group. Sham procedures involved exposure and manipulation of the stomach. After surgery, mice were fed a liquid diet Osmolite 1Cal (Abbott Nutrition, Columbus, OH) for 4 days, then switched into either ND or HFD for 8 weeks.

### Immunofluorescence

Whole-mount adipose tissue explants were fixed overnight in a 1% paraformaldehyde solution (Electron Microscopy Sciences, Hatfield, PA) and were used for immunofluorescence as previously described [8]. Antibodies used for immunofluorescence included Caveolin1 (Polyclonal rabbit-anti-Caveolin, BD Pharmingen, San Jose, CA), Mac2 (Rat Galectin-3 monoclonal antibody (M3/38), BD Pharmingen, San Jose, CA), and 4′,6-Diamidine-2′-phenylindole dihydrochloride (DAPI) (Roche, Basel, Swiss). Secondary antibodies included goat anti-rabbit IgG Alexa Fluor 488 (polyclonal, Invitrogen, Carlsbad, CA) and goat anti-rat IgG Alexa Fluor 568 (polyclonal, Invitrogen, Carlsbad, CA).

### Flow cytometry

Adipose tissue and liver were digested in RPMI 1640 (Gibco, Indianapolis, IN) with 1 mg/ml collagenase (Clostridiopeptidase A from *Clostridium histolyticum* type II Sigma-Aldrich, St. Louis, MO) as previously described [22] on a rocking platform shaker for 25 min at 37 °C. The stromal vascular fraction (SVF) was separated from adipocytes by centrifugation. The following antibodies were used for flow cytometry: anti-mouse CD45 eFluor 450 (30-F11 monoclonal, Invitrogen, Carlsbad, CA), anti-mouse CD11c eFluor 780 (N418 monoclonal, Invitrogen, Carlsbad, CA), anti-mouse CD64 PE (X54-5/7.1 monoclonal, BD Pharmingen, San Jose, CA), and anti-mouse Ly6G (Gr1) FITC (IA8, BD Pharmingen, San Jose, CA). Adipose tissue macrophages are CD64^+^ and separated as M2 (CD11c^−^) or M1 (CD11c^+^). Dendritic cells are CD64^−^CD11c^+^ cells. Analysis was performed using a BD Biosciences FACSAria and FlowJo v.10 (Treestar) software.

### Statistical analysis

Results are presented as mean ± SEM. The analysis was first performed to evaluate for any significant main effects and interactions regarding sex and diet. Comparisons were then made with one-way ANOVA using Tukey’s method followed by a three-factor general linear model. Table 1 provides the results of general linear models assessing the significance of the main effects and interactions for the three factors: sex, surgery, and diet. Table 2 gives the results of Tukey’s multiple comparisons by providing adjusted *p* values.

**Table 1:** Significance of main effects and interactions (results for VSG experiment)

Variables	*P* values for						
	Sex (M/F)	Surgery (V/S)	Diet (C/H)	Sex and surgery	Sex and diet	Surgery and diet	Three factors
Size parameters							
Weight	*0.000*	*0.000*	*0.000*	*0.049*	*0.000*	0.116	0.658
GWAT mass	*0.000*	*0.004*	*0.000*	0.945	*0.000*	0.141	0.404
IWAT mass	*0.000*	*0.000*	*0.000*	*0.049*	*0.000*	*0.001*	0.063
Liver mass	*0.000*	*0.005*	*0.000*	*0.005*	*0.001*	*0.010*	*0.012*
Spleen mass	*0.045*	*0.000*	*0.000*	0.067	0.238	0.445	0.262
GWAT Adipocyte size	0.367	*0.005*	*0.000*	0.536	*0.014*	–	
GWAT leukocytes							
GWAT ATM	*0.000*	0.466	*0.000*	*0.035*	*0.034*	*0.015*	0.490
GWAT CD11c^+^	*0.000*	*0.000*	*0.011*	*0.015*	0.154	0.676	0.452
GWAT CD11c^−^	*0.000*	0.053	*0.000*	0.306	0.061	*0.002*	0.717
GWAT DC	0.682	0.418	0.596	*0.043*	0.181	0.647	0.608
GWAT PMN	0.387	*0.000*	0.958	0.201	0.889	0.833	0.886
IWAT leukocytes							
IWAT ATM	0.248	0.801	*0.000*	0.147	0.162	–	
IWAT CD11c^+^	0.783	*0.003*	*0.000*	*0.005*	*0.000*	–	
IWAT CD11c^−^	0.201	0.667	*0.002*	0.308	0.496	–	
IWAT DC	*0.010*	*0.008*	*0.000*	0.363	0.051	–	
Liver leukocytes							
Liver TM	0.315	*0.000*	0.284	0.918	*0.026*	0.187	0.325
Liver TM CD11c^+^	0.278	0.606	*0.000*	*0.021*	*0.000*	0.858	*0.024*
Liver TM CD11c^−^	0.226	*0.000*	0.600	0.770	0.081	0.168	0.508
Liver DC	*0.035*	*0.004*	*0.000*	*0.000*	*0.000*	*0.001*	*0.000*
Liver PMN	0.156	*0.000*	0.816	0.196	0.745	0.579	0.163

**Table 2:** Comparison of diet groups by gender. Adjusted *p* values for Tukey’s simultaneous tests. Surgery comparisons are shown in Figs. 5, 6, 7, and 8 (results for VSG experiment)

Variable	Surgery comparisons				Diet comparisons				Sex comparisons			
	MSC-MVC	MSH-MVH	FSC-FVC	FSH-FVH	MSC-MSH	MVC-MVH	FSC-FSH	FVC-FVH	MSC-FSC	MSH-FSH	MVC-FVC	MVH-FVH
Size parameters												
Weight	0.590	*0.000*	1.000	0.618	*0.000*	*0.000*	*0.001*	0.101	*0.001*	*0.000*	0.572	*0.000*
GWAT mass	0.979	0.388	1.000	0.081	*0.000*	*0.000*	*0.000*	0.046	0.817	*0.000*	1.000	1.000
IWAT mass	1.000	*0.000*	1.000	0.429	*0.000*	*0.000*	*0.002*	0.400	1.000	*0.000*	1.000	*0.000*
Liver mass	1.000	*0.000*	1.000	1.000	*0.000*	0.890	0.997	0.999	0.306	*0.000*	0.894	0.052
Spleen mass	0.111	*0.000*	0.223	0.257	0.252	*0.025*	0.431	0.780	1.000	1.000	0.999	*0.011*
Adipocyte size	–	0.160	–	0.532	*0.000*	–	0.053	–	0.979	0.086	–	0.474
GWAT leukocytes												
GWAT ATM	1.000	*0.010*	0.792	1.000	*0.000*	0.876	0.462	1.000	*0.018*	*0.000*	0.907	*0.012*
GWAT CD11c^+^	0.407	*0.001*	0.995	1.000	*0.045*	0.924	1.000	0.996	*0.001*	*0.000*	0.719	*0.033*
GWAT CD11c^−^	0.544	0.762	0.112	1.000	*0.000*	0.982	0.191	1.000	0.874	*0.004*	1.000	0.249
GWAT DC	0.917	0.696	0.965	1.000	0.956	0.991	1.000	0.988	0.985	0.577	0.876	1.000
GWAT PMN	0.205	*0.005*	0.744	0.774	1.000	1.000	1.000	1.000	1.000	1.000	0.981	0.797
IWAT leukocytes												
IWAT ATM	–	0.850	–	0.941	*0.007*	–	0.571	–	0.862	*0.038*	–	0.991
IWAT CD11c^+^	–	*0.003*	–	1.000	*0.000*	–	1.000	–	0.999	*0.000*	–	0.988
IWAT CD11c^−^	–	0.999	–	0.881	0.064	–	0.446	–	0.725	0.206	–	0.994
IWAT DC	–	0.146	–	0.727	*0.000*	–	0.156	–	0.760	*0.001*	–	0.237
Liver leukocytes												
Liver TM	*0.031*	0.747	0.194	0.155	*0.025*	1.000	1.000	0.996	0.935	0.240	1.000	0.909
Liver TM CD11c^+^	1.000	*0.039*	1.000	0.404	*0.000*	0.316	0.926	0.986	0.132	*0.002*	0.516	0.960
Liver TM CD11c^−^	*0.014*	0.324	0.124	0.224	0.184	1.000	1.000	0.984	0.991	0.575	1.000	0.799
Liver DC	1.000	*0.000*	1.000	0.982	*0.000*	0.177	0.823	0.607	0.788	*0.000*	0.951	0.999
Liver PMN	*0.009*	0.298	0.958	0.621	0.996	0.828	1.000	0.999	1.000	0.999	0.279	0.998

## Results

### Males have impaired metabolism with HFD that resolves in weight loss (WL), while females remain metabolically intact

**Fig. 1:**
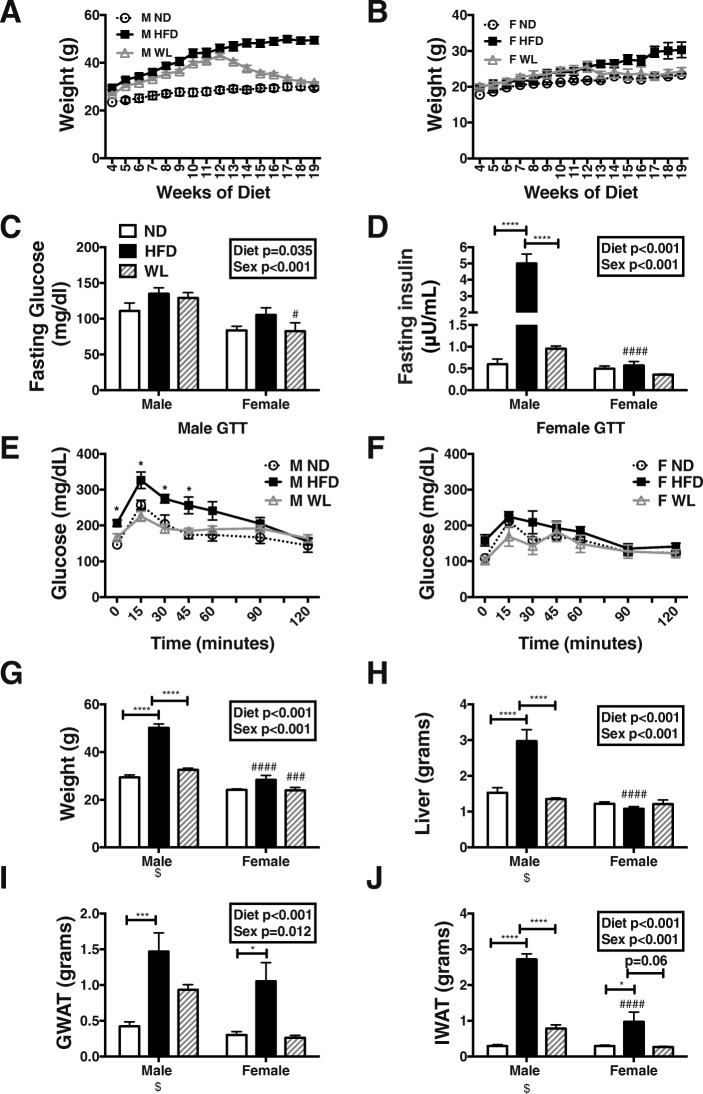
Males specifically have impaired metabolism with high-fat diet exposure that resolves with weight loss. WT male and female C57Bl/6J mice were placed on high-fat diet (HFD) starting at 6 weeks of age. After 12 weeks of diet, a group of these mice was switched to normal chow to mimic dietary weight loss (WL). A control group remained on normal chow the entire time (ND). Weekly weights were recorded in males (**a**) and females (**b**). Metabolic studies included **c** fasting glucose and **d** fasting insulin at 6 weeks of WL and glucose tolerance (GTT) testing at 7 weeks of WL in **e** males and **f** females. At 26 weeks of age, animal **g** weight and tissue weight including **h** liver, **i** gonadal white adipose tissue (GWAT), and **j** inguinal white adipose tissue (IWAT). Statistics from diet and sex interaction in box*p < 0.05, ****p < 0.001. ###*p* < 0.005 and ####*p* < 0.001 for comparisons between sexes of the same diet group. *N* = 4–8 per group. $ Data previously published [23]

Male and female C57Bl/6J mice were placed on ND or fed a 60% HFD starting at 6 weeks of age. After 12 weeks of HFD, one group of animals was switched back to a control chow to mimic dietary WL. After 8 weeks of normal chow, the body weights of the WL group reduced and were similar to the ND animal weights for both males and females (Fig. 1a, b). After 6 weeks of WL, all animals were fasted for 6 h and serum glucose and insulin evaluated. Fasting glucose levels varied by diet in both sexes and were significantly lower in WL females compared to males (*p* < 0.001 for diet and sex effects) (Fig. 1c). Male insulin levels were increased with HFD, as we have previously shown [11], but female insulin levels did not significantly increase with HFD and were significantly lower in HFD females (Fig. 1d). After just 6 weeks, the WL group had significantly lower insulin levels in male mice (Fig. 1d). At 19 weeks of HFD, glucose tolerance testing was impaired in males. However, with 7 weeks of diet switch, glucose tolerance improved in male WL animals compared to HFD (Fig. 1e). Female animals remained glucose tolerant in HFD and WL states (Fig. 1f).

As expected, HFD increased body mass and liver mass in males and weight loss reduced both body weight and liver weight to that of control animals, as previously published [23] (Fig. 1g, h). There was a significant sex effect with female HFD animals having lower weight and liver mass (Fig. 1g, h). HFD animals had increased visceral gonadal white adipose tissue (GWAT) and subcutaneous inguinal white adipose tissue (IWAT) depots in both sexes (Fig. 1i, j). After 8 weeks of diet switch, WL animals still had heavier GWAT fat pads while IWAT weights returned to control weights in both sexes (Fig. 1i, j).

### Males have an increased myeloid response to HFD that persists in WL

**Fig. 2:**
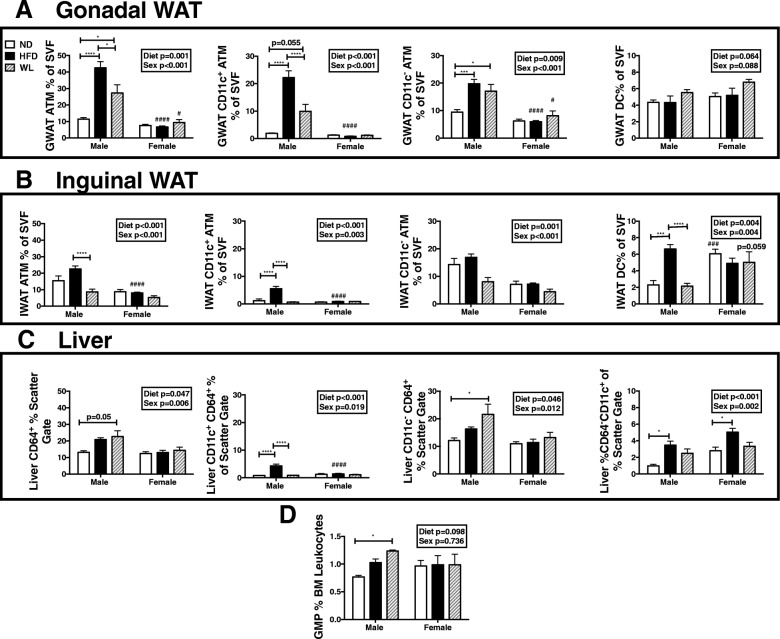
Males have increased tissue macrophage responses in high-fat diet that persist in weight loss. WT male and female C57Bl/6J mice were placed on high-fat diet (HFD) starting at 6 weeks of age. After 12 weeks of diet, a group of these mice was switched to normal chow to mimic dietary weight loss (WL). A control group remained on normal chow the entire time (ND). Stromal vascular fractions were isolated and adipose tissue macrophages (ATM) and dendritic cells (DCs) assessed by flow cytometry in **a** GWAT, **b** IWAT, and **c** tissue macrophages and DCs in the liver. **d** Bone marrow granulocyte-macrophage progenitors (GMPs) were also evaluated by flow cytometry. Statistics from diet and sex interaction in box. *p < 0.05, ***p < 0.005, ****p < 0.001. #*p* < 0.05, ### *p* < 0.005, and ####*p* < 0.001 for comparisons between sexes of the same diet group. *N* = 4–8 per group

To assess potential changes in the degree of inflammation after a WL intervention, we isolated targeted metabolic tissues and evaluated leukocytes by flow cytometry in the weight loss mice model. GWAT macrophages expanded in HFD males and did not fully resolve in WL mice (Fig. 2a). HFD-fed females had no expansion in GWAT macrophages, and these did not change with diet switch (Fig. 2a). In males, GWAT ATMs regardless of CD11c type were increased in HFD and WL, though weight loss did show inflammatory CD11c^+^ ATM reduction when compared to HFD animals (Fig. 2a). Dendritic cells (DCs) were similar amongst all groups in both sexes (Fig. 2a).

A similar pattern of expansion of ATMs was seen in male HFD IWAT, but DCs were also expanded and both myeloid populations reduced with WL (Fig. 2b). ATMs and DCs in subcutaneous inguinal fat did not significantly expand in HFD females and were unchanged with weight loss (Fig. 2b). Tissue macrophages increased within the liver in males on HFD (Fig. 2c). Within the liver, CD64^+^ cells increased only marginally in males with HFD but significantly so with WL and were mostly the CD11c^−^ type. CD64^−^CD11c^+^ cells increased with HFD in both sexes and reduced with WL (Fig. 2c). Similar to the fat depots, WL resolved CD11c^+^CD64^+^ cell accumulation in male livers as well. The resolution in CD11c^+^ populations is critical given that this cell population is strongly associated with antigen presentation and T cell activation. Since CD11c^+^ ATMs are thought to be recruited, it is interesting that this macrophage population completely improved in IWAT and liver, but not in GWAT.

Consistent with our prior studies, myeloid granulocyte and macrophage precursors (GMPs) increased in males with HFD but more significantly so in WL [8, 24] (Fig. 2d). Overall, these results demonstrate that in males, myeloid inflammation is only partially improved with WL in visceral tissues but significantly improved in liver and IWAT.

### Hematopoietic cells transplanted from WL animals are not primed to impair metabolism

**Fig. 3:**
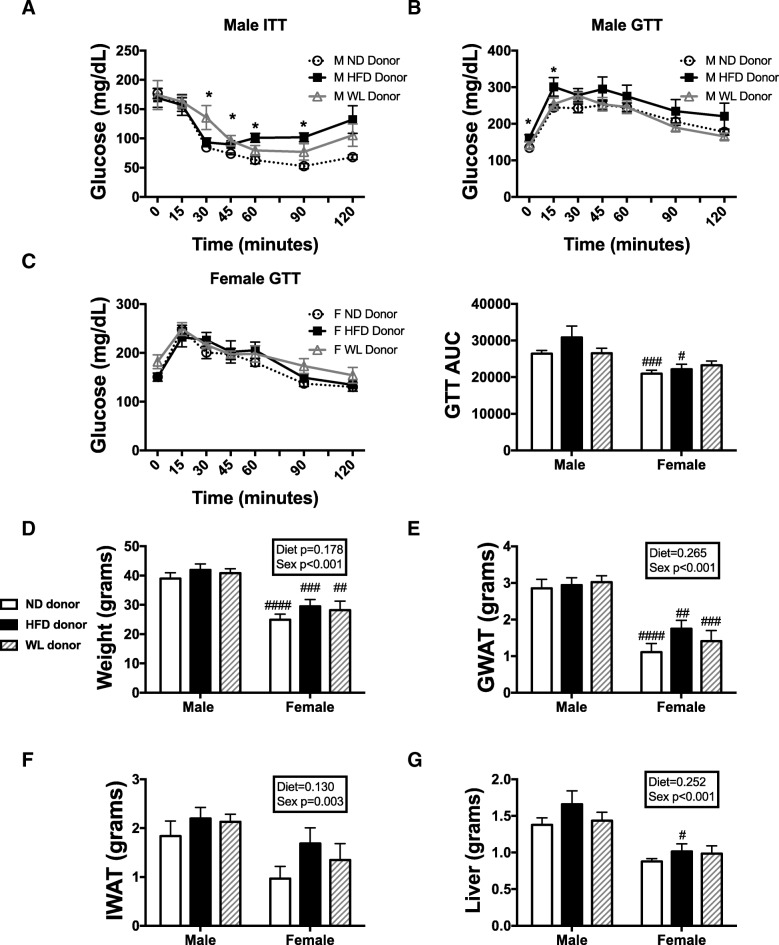
Male and female hematopoietic cells transplanted from weight loss animals are no longer primed to impair metabolism. ND, HFD, and WL animals were generated and at 26 weeks of age, bone marrow was injected into irradiated C57Bl6/j mice of the same sex. Six weeks following bone marrow transplantation, all mice were started on a 60% HFD. **a** Insulin tolerance tests (ITT) studies were performed in males at 14 weeks of HFD. GTT studies were performed at 12 weeks of HFD in **b** males and **c** females with calculated area under the curve (AUC). After 16 weeks of HFD, animal **d** weight and tissue, **e** GWAT, **f** IWAT, and **g** liver were measured. *p < 0.05. #*p* < 0.05, ##*p* < 0.01, ### *p* < 0.005, ####*p* < 0.001 for comparisons between sexes of the same diet group. *N* = 4–10 per group

To determine whether hematopoietic stem cell changes due to obesity or weight loss might contribute to varied inflammatory responses, we performed bone marrow transplant experiments. Twenty-six-week-old animals from ND, HFD, or WL groups were used as bone marrow donors. Bone marrow was isolated and injected into 8-week-old WT C57Bl/6J irradiated recipient animals. For these experiments, donor sex was the same as recipient sex. Animals were allowed to recover from bone marrow transplantation (BMT), and 6 weeks post-BMT were challenged to 16 weeks HFD to determine if a subsequent HFD challenge, second hit, to the bone marrow would lead to a priming for increased metabolic inflammation. In males, we have previously observed that with 16 weeks of HFD, bone marrow transplant and re-challenge leads to increased glucose impairment over ND recipient BMT animals [8]. Consistent with prior results, males with HFD donor BM had impaired insulin sensitivity as seen in insulin tolerance test (ITT) (Fig. 3a) and glucose intolerance in males (Fig. 3b). While not significant, male animals with WL BM tended to have higher glucoses in response to insulin (ITT) (*p* = 0.07 ND vs WL).

**Fig. 4:**
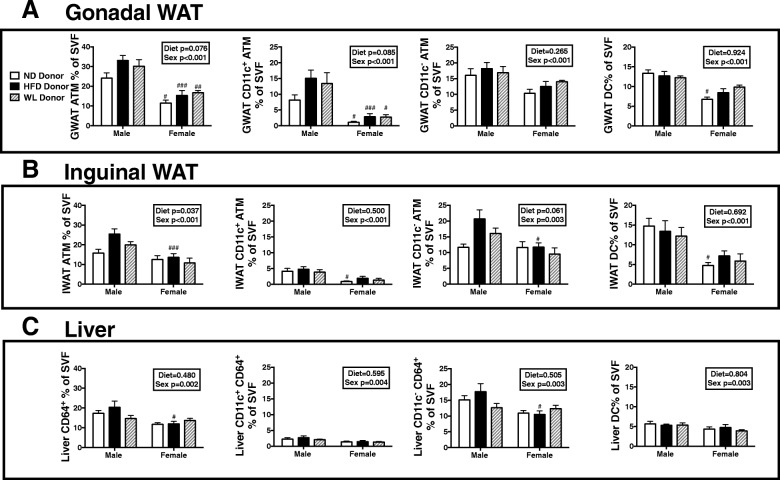
Male HFD donor marrow generates more ATMs even post-BMT. ND, HFD, and WL animals were generated and at 26 weeks of age bone marrow was injected into irradiated C57Bl6/j mice of the same sex. Six weeks following bone marrow transplantation, all mice were started on a 60% HFD. **a** GWAT, **b** IWAT, and **c** liver tissue macrophages, subsets, and DCs were measured. #*p* < 0.05, ##*p* < 0.01, ### *p* < 0.005, ####*p* < 0.001 for comparisons between sexes of the same diet group. *N* = 4–10 per group

In contrast, no changes were observed in female groups regardless of donor group (Fig. 3c). While there were differences in glucose homeostasis in males, there was no significant difference in body weight (Fig. 3d), GWAT (Fig. 3e), IWAT (Fig. 3f), or liver (Fig. 3g) by donor group. This suggests that the HFD donor BM impaired glucose homeostasis independent of body, fat pad, or liver mass. As expected, weights for all variables were lower in females than males (sex effect *p* < 0.001). We further isolated tissues to look at tissue macrophages and found that there was a near significant diet effect and animals with male HFD donor marrow had a larger number of ATMs in GWAT and IWAT (Fig. 4a, b) and more total ATMs than female mice (sex effect *p* < 0.001). There were no significant differences in GWAT or IWAT ATM type or DCs, although females still had lower numbers (Fig. 4a, b). A similar trend was observed in the liver with lower numbers of macrophages in females but no differences amongst diet groups (Fig. 4c). Overall, in female animals, marrow, regardless of prior exposure, remains protected from myeloid responsiveness to HFD.

### VSG and WL both improve body composition of formerly HFD fed mice regardless of sex

**Fig. 5:**
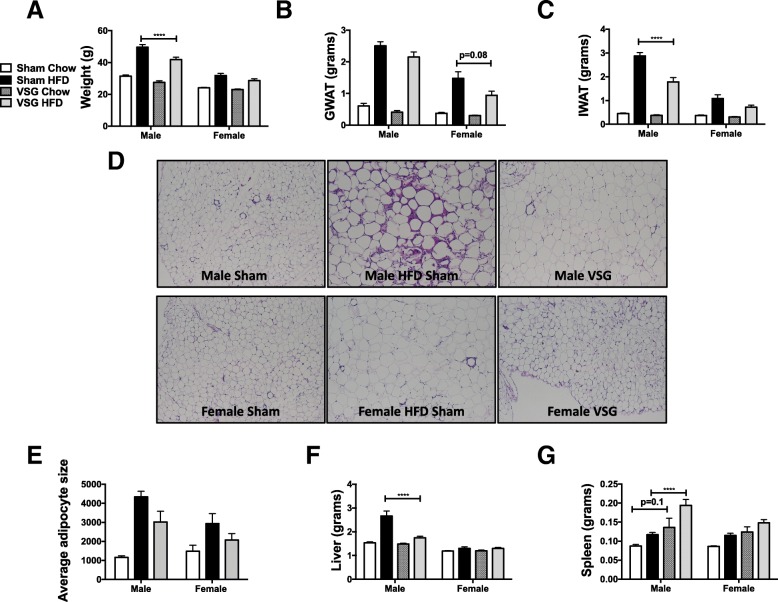
VSG improves body composition but less than switch to standard chow. Six-week-old C57Bl/6j males and females were fed HFD for 12 weeks. At that time, animals underwent sham surgery or vertical sleeve gastrectomy (VSG). Sham animals were either switched to chow or remained on HFD for an additional 8 weeks. At 26 weeks of age, animals were sacrificed and **a** total weight, **b** GWAT weight, and **c** IWAT weight recorded. **d** GWAT H&E slides were imaged (**e**) and adipocyte sizing was performed. **f** Liver and **g** spleen weights were also recorded. *p < 0.05, ***p < 0.005, ****p < 0.001. #*p* < 0.05, significant differences are shown only between sham chow and VSG chow or sham HFD or VSG HFD *N* = 6–9 for VSG chow groups, *N* = 7–11 for adipocyte sizing, and *N* = 13–18 for all other data per group

To understand whether surgically induced weight loss responses differ from standard diet weight switch, animals were treated with 12 weeks of HFD and then underwent sham or VSG surgery. Post-surgery, sham animals were either maintained on HFD or switched to a standard chow, to mimic WL controls. VSG animals were standardly maintained on HFD to assess surgery weight loss effects alone. A small group of VSG animals was switched to standard chow after surgery to mimic our prior WL studies. Chow switch in sham and VSG animals led to improved weight loss compared to VSG alone (Fig. 5a), with greater improvement in fat mass loss (Fig. 5b, c) and reduction in adipocyte size changes (Fig. 5d, e). VSG itself without diet switch led to improved body weight and IWAT weight in males (Fig. 5a, c), with near significant improvement in GWAT weight in females (Fig. 5b). However, liver weight increased only in male HFD-fed animals and resolved in both VSG treatment groups or chow switch. No effects of liver weight were observed in either of the female groups (Fig. 5f). Overall, while VSG improved body weight, IWAT, and liver mass, there was an additive effect with chow switch. Interestingly, splenomegaly occurred in VSG groups of both sexes but significantly so only in male HFD groups (Fig. 5g).

### VSG and WL both decrease obesity-associated adipose tissue CD11c^+^ ATMs in male mice

**Fig. 6:**
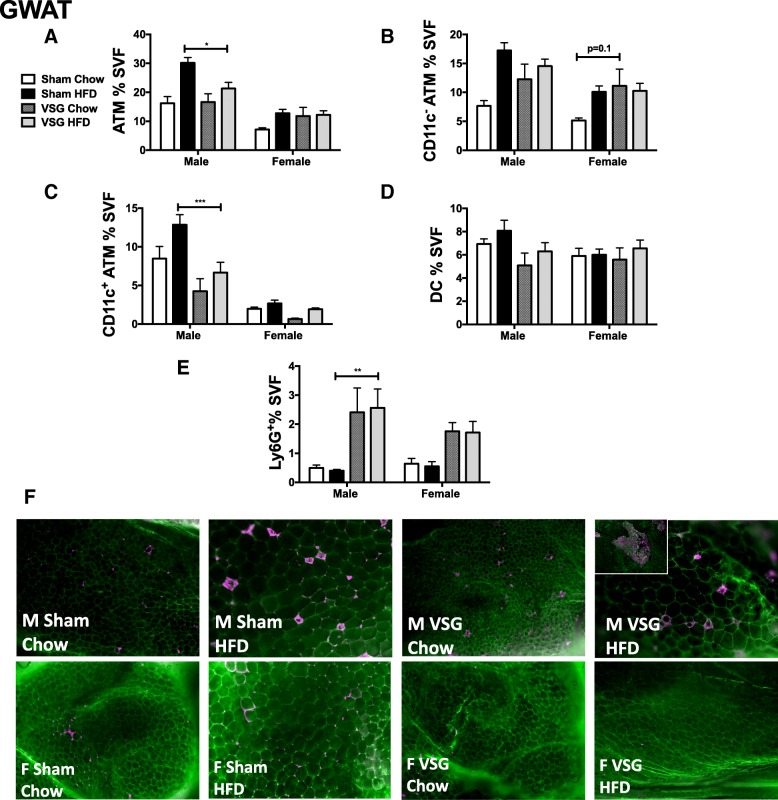
VSG improves CD11c^+^ adipose macrophages but CD11c^−^ ATMs do not fully recover in both sexes. Six-week-old C57Bl/6j males and females were fed HFD for 12 weeks. At that time, animals underwent sham surgery or vertical sleeve gastrectomy (VSG). Sham animals were either switched to chow or remained on HFD for an additional 8 weeks. At 26 weeks of age, animals were sacrificed and GWAT stromal vascular fraction (SVF) was isolated and flow cytometry used to quantitate **a** ATMs, **b** CD11c^−^ ATMs, **c** CD11c^+^ ATMs, **d** dendritic cells (DC), and **e** neutrophils (Ly6G^+^ cells). **f** Immunofluorescence performed with caveolin (green) and Mac2 (purple) staining. Inset demonstrating areas of clustered macrophages seen in male VSG samples. *p < 0.05, ***p < 0.005, ****p < 0.001, significant differences are shown only between sham chow and VSG chow or sham HFD or VSG HFD. *N* = 6–9 for VSG chow groups, *N* = 13–18 for ATM and DC populations, and *N* = 5–10 for adipose neutrophils

**Fig. 7:**
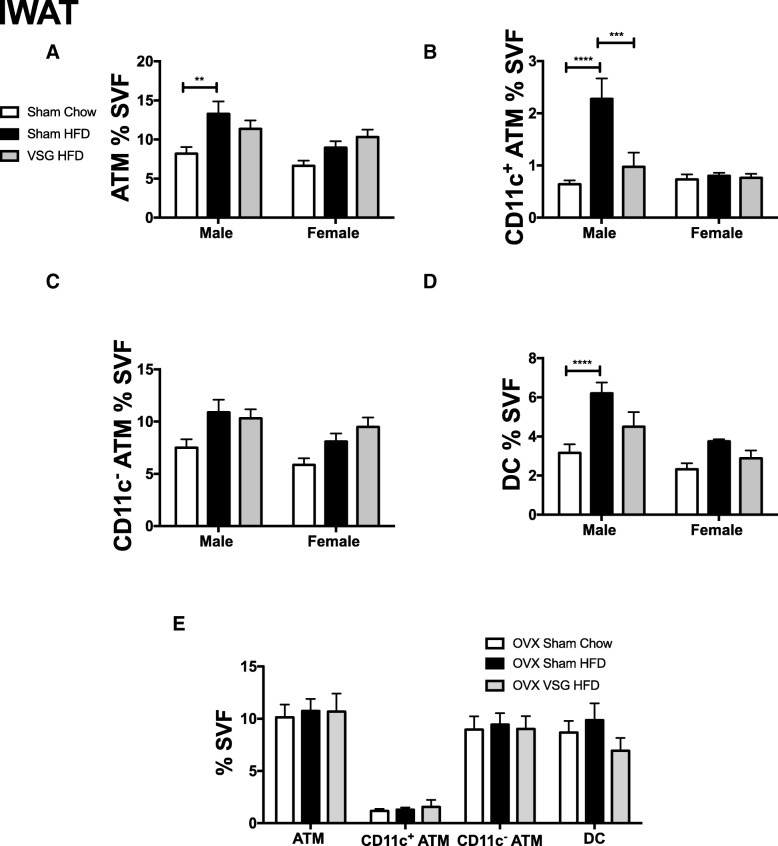
VSG improves IWAT CD11c^+^ ATMs and DCs. Six-week-old C57Bl/6J males and females were fed HFD for 12 weeks. At that time, animals underwent sham surgery or vertical sleeve gastrectomy (VSG). Sham animals were either switched to chow or remained on HFD for an additional 8 weeks. At 26 weeks of age, animals were sacrificed and IWAT stromal vascular fraction (SVF) was isolated and flow cytometry used to quantitate **a** ATMs, **b** CD11c^−^ ATMs, **c** CD11c^+^ ATMs, and **d** dendritic cells (DC). **e** Myeloid leukocyte subtypes profiled in ovariectomized female mice. *p < 0.05, **p < 0.01, ****p < 0.001. *N* = 9–18

Flow cytometry analysis of visceral adipose tissue (VAT) macrophages showed that a chow switch reduced ATMs and to a greater extent than VSG in males and females (Fig. 6a). A switch to chow significantly decreased ATMs when comparing both subtypes (CD11c^+^ and CD11c^−^) (Fig. 6b, c), while VSG decreased mostly CD11c^+^ ATMs. DCs were also reduced but not significantly different with chow switch and VSG in male animals (Fig. 6d). VSG however increased neutrophils indicated by Ly6G^+^ cells in adipose tissue in both sexes with lack of significant two-factor interaction but significant surgery effect (*p* < 0.001) (Table 1 and Fig. 6e). The alteration in CD11c^+^ ATM accumulation was seen by persistent CLS in male animals in sham HFD, with some improvement with VSG (Fig. 6f). The CLSs appear more prominent in this group due to continued adipocyte hypertrophy in HFD-fed animals while adipocytes were small with the chow switch (Fig. 6f). Similar findings were observed in the IWAT compartment, with greater reduction in CD11c^+^ ATMs by chow switch in males only (Fig. 7a, b). Unlike the GWAT, CD11c^−^ ATMs and DCs did not show any significant changes in the VSG HFD groups in male and female IWAT (Fig. 7c, d). To understand if the female protection from adipose inflammation was due to estrogen, a subgroup of ovariectomized mice underwent VSG and was found to have little change in ATM accumulation with no significant change in inflammation with VSG (Fig. 7e).

### VSG resolves pro-inflammatory ATMs in males but promotes CD11c^−^ TMs numbers in both sexes in liver

**Fig. 8:**
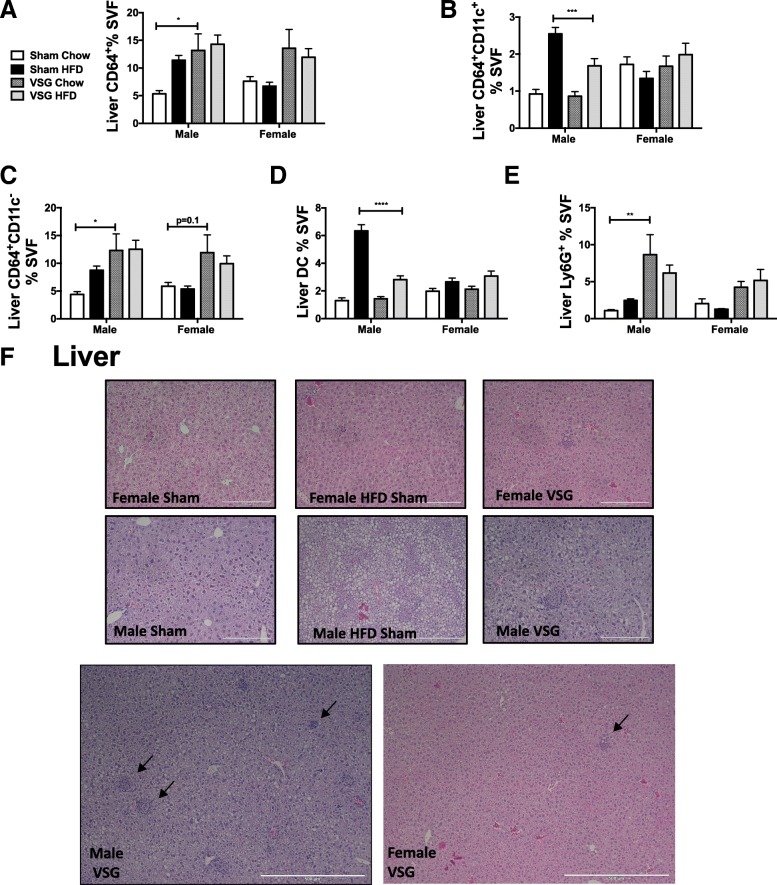
VSG increases liver myeloid cells in both sexes. Six-week-old C57Bl/6J males and females were fed HFD for 12 weeks. At that time, animals underwent sham surgery or vertical sleeve gastrectomy (VSG). Sham animals were either switched to chow or remained on HFD for an additional 8 weeks. At 26 weeks of age, animals were sacrificed and liver stromal vascular fraction (SVF) was isolated and flow cytometry used to quantitate **a** macrophages, **b** CD11c^−^ macrophages, **c** CD11c^+^ macrophages, **d** dendritic cells (DC), and **e** neutrophils (Ly6G^+^ cells). **f** Liver, a H&E demonstrating areas of extramedullary hematopoiesis. *p < 0.05, **p < 0.01, ***p < 0.005, ****p < 0.001, significant differences are shown only between sham chow and VSG chow or sham HFD or VSG HFD. *N* = 6–9 for VSG chow groups, *N* = 13–18 for macrophages and DC populations and *N* = 5–10 for neutrophils

VSG is well known to relieve obesity-related hyperlipidemia and NAFLD [25]. In addition to steatosis, liver inflammation and fibrosis is part of the pathology to steatohepatitis. Therefore, we next evaluated leukocytes within the liver. In males, HFD enhances CD64^+^ cells with some resolution with chow switch but not with VSG (Fig. 8a). While CD64^+^CD11c^+^ cells increased with HFD in males (as seen in Fig. 2c), they improved both with VSG and even more with chow switch. CD64^+^ CD11c^−^ cells, however, increased with VSG (Fig. 8b, c). DCs were also expanded with HFD but improved with VSG and chow switch in males (Fig. 8d). Females failed to show any significant effects with VSG on liver macrophages (Fig. 8a–d). Imaging studies showed dense pockets of leukocytes suggesting extramedullary hematopoiesis in post-VSG animals (Fig. 8f) that corresponded with increased neutrophils by flow cytometry in both sexes as demonstrated by lack of significant two-factor interaction but significant surgery effect (*p* < 0.001) (Table 1 and Fig. 8e).

## Discussion

Our findings demonstrate that weight loss intervention strategies have a more profound effect in myeloid inflammatory reduction in males than females. This observation holds true for both dietary-related weight loss and VSG. It is worth noting that females are protected from obesity-induced myeloid inflammatory responses relative to males and overall have a decreased capacity to elicit a myeloid reduction. Nevertheless, our results suggest that both weight loss intervention strategies are more effective in reducing pro-inflammatory myeloid cell populations in males than females. However, both the strategies of dietary and surgical interventions to reduce obesity failed to completely resolve myeloid inflammation in male mice, although inflammation was lowered more with dietary intervention. This is crucial because it shows that dietary strategies should be considered in addition to surgical intervention alone, in males. It also suggests that some of the benefits attributed to surgery are dependent on an improved dietary intake after surgery. When utilized independently, both intervention strategies should be considered only semi-effective strategies in reducing myeloid cell populations in males, although metabolism is improved.

Surprisingly, there was a higher level of CD11c^−^ ATMs with VSG in GWAT of both sexes although CD11c^+^ ATMs improved (Fig. 6). Ly6G^hi^ neutrophil populations were exacerbated by VSG and may likely be correlated with an increase in splenic mass and extramedullary hematopoiesis in both sexes (Fig. 5g). CD11c^+^ populations, which have been highly implicated in metabolic disease, are affected differentially by intervention strategy. It is possible that the type of weight loss has a significant difference in metabolic disease, with standard dietary weight loss improving myeloid inflammation compared to VSG/surgical weight loss due to the slower nature of weight loss with diet versus surgery. Also, all animals in the surgical groups with either sham or VSG surgery had generally lower weights and lower ATM content than just WL experiments. This could be due to recovery from a surgery or from expansion of another population of SVF cells (leukocytes or pre-adipocytes) that was not measured in these experiments.

Weight loss programs are primarily undertaken in women, but the impact on inflammation and metabolism is more significant in males since weight loss by both dietary and surgical interventions reduced metabolic impairment and inflammation more in males. Clinically, it has been found that males and females have different metabolic responses to low energy diet, with men having greater reductions in metabolic syndrome *Z* score, C-peptide, fat mass, and heart rate, while females had greater reductions in HDL cholesterol, blood pressure, and hip circumference [26]. Animal studies have previously demonstrated that in males, weight loss specifically leads to improved metabolism; however, inflammation persists due to local macrophage proliferation [11]. Mechanistically, this is thought to be due to enhanced lipolysis promoting proliferation of immune cells [27]. Although female animals also have significant adiposity, improvements yet fail to generate inflammation with standard weight loss suggesting that there is a sex difference in response to this type of slow lipolysis. One possibility is that females have greater capability to store adipose tissue and therefore a lesser requirement to generate ATMs that are important for clearance of fatty acids and lipids. Also, female ATMs may have a greater potential for beta oxidation not requiring pro-inflammatory activation. Hence, a sex difference in weight loss mediated lipolytic response and lipid metabolism may contribute to sex differences in inflammation. Interestingly, recent studies performed in rats have shown a similar increased inflammatory phenotype in female livers post-VSG associated with worsened hepatic triglycerides [28]. The findings of extramedullary hematopoiesis and likely myeloid progenitor increases even after weight loss and BMT are likely mechanisms causing such persistent inflammation in WL even in a clinical setting.

Unlike standard WL, during VSG, both sexes exhibit some splenomegaly and myeloid inflammation increases with neutrophils in the GWAT and myeloid cells in the liver. This suggests that with acute lipolysis, both sexes are primed to produce inflammation to respond to the rapid weight loss, but the response is greater in male animals. Previously, similar systemic inflammatory responses were demonstrated in male animals [12]. While our finding in females is unexpected, it is a clinically relevant result since nearly 80% of bariatric surgery patients are females [14] and success in weight reduction and T2DM resolution has been observed 5 years after VSG in predominantly female cohorts [29]. However, it is critical to continue to monitor risk for meta-inflammation and its cardiovascular and metabolic consequences in females [30].

Men have been shown to have increased morbidity and resource utilization post-bariatric surgery [31], and our findings of persistent inflammation in male mice may explain some of this result. One limitation of our study is the identification of the key factors that promote the systemic inflammatory response post-VSG. We did observe that these factors are related to the VSG surgery itself, and not related to a foreign body response, using a staple sham group (not shown). There are many possible lipid mediators, inflammatory mediators, and altered hormones that are likely be responsible, but given systemic hematopoiesis, it is possible that there are factors systemically released that drive myelopoiesis or that prompt the need for inflammatory leukocytes. Specifically, free fatty acids may directly stimulate ATM proliferation [27, 32]. However, this release from tissues was not specifically measured in these studies but many lipid mediators are known to trigger both ATM proliferation and hematopoiesis [32, 33]. Additionally, given that there is significant adipose remodeling and there are likely hematopoietic progenitors within adipose tissue, it may be this reservoir that remains activated in males even post-weight loss [34]. Another limitation of our findings is that female animals did not have significant obesity although they gain weight with HFD challenge. It is possible that this is due to the use of the C57Bl/6J strain which is commonly used in obesity but does not have the same obesity induction in females as other strains such as DBA/2J and C3H/HeJ. However, these two strains do not have as robust of an inflammatory response. Future studies in multiple strains with both sexes are necessary to understand the generalizability of these results.

A unique aspect of our studies is the evaluation of tissue-specific leukocytes in both VSG and standard dietary WL. Clinical studies using RNASeq of adipose tissue have shown improved inflammatory markers after VSG [35], but a major limitation of these studies is that they were performed only in females. However, in these studies, there are some persistent inflammatory signals of interferon signaling pathways suggesting that some tissue remodeling continues post dietary weight loss [36] and VSG especially in those with higher insulin resistance [37, 38]. The ATM profile changes we see in our study hence have a similar phenotype to the clinical models of weight loss. A limitation to our study is that sometimes as obese men and women undertake lifestyle changes, they also increase physical activity. It is possible that if individuals were to further alter their lifestyle with diet and exercise, they may extend the improvement in inflammation compared to surgery or dietary-induced weight loss alone. Exercise independently reduces inflammation with more M2 macrophages and reduced total myeloid in adipose tissue [39] and liver [40]. Further investigations into the effects of combined weight loss strategies are necessary to optimize the metabo-inflammatory responses to weight loss.

Overall, these results emphasize that sex differences in inflammatory responses to high-fat diet and weight loss make it critical to track prior weight history when counseling on metabolic and cardiovascular risk factors in the formerly obese individual. These results also suggest the importance of evaluating inflammation prior to and after weight loss interventions particularly in men. While we have not identified all the factors that need to be considered with weight loss intervention, we have shown that factors such as the method of weight loss, gender, and disease risks are important when caring for previously obese patients and when monitoring for any inflammation related conditions.

## Abbreviations

ATM: Adipose tissue macrophage
GMP: Granulocyte-monocyte progenitor
GTT: Glucose tolerance test
GWAT: Gonadal white adipose tissue
HFD: High-fat diet
HSC: Hematopoietic stem cells
IWAT: Inguinal white adipose tissue
ND: Normal diet
VAT: Visceral adipose tissue
VSG: Vertical sleeve gastrectomy
WL: Weight loss
